# Isolation of A Novel *Bacillus thuringiensis* Phage Representing A New Phage Lineage and Characterization of Its Endolysin

**DOI:** 10.3390/v10110611

**Published:** 2018-11-06

**Authors:** Yihui Yuan, Qin Peng, Shuo Yang, Shaowen Zhang, Yajuan Fu, Yan Wu, Meiying Gao

**Affiliations:** 1 State Key Laboratory of Marine Resource Utilization in South China Sea, Hainan University, Haikou 570228, China; yuanyh@hainu.edu.cn (Y.Y.); yangshuohainu@126.com (S.Y.); xiaoying_zhang@163.com (S.Z.); 2 Wuhan Institute of Virology, Chinese Academy of Sciences, Wuhan 430071, China; fuyajuann@163.com (Y.F.); wuyan_81@126.com (Y.W.); 3 Ministry of Education Key Laboratory for Ecology of Tropical Islands, College of Life Sciences, Hainan Normal University, Haikou 571158, China; pengqin1019@126.com

**Keywords:** bacteriophage, endolysin, restriction modification system, *Bacillus thuringiensis*, tadpole-like phage virion

## Abstract

Phages, the parasites of bacteria, are considered as a new kind of antimicrobial agent due to their ability to lyse pathogenic bacteria. Due to the increase of available phage isolates, the newly isolated phage showed increasing genomic similarities with previously isolated phages. In this study, the novel phage vB_BthS_BMBphi, infecting the *Bacillus thuringiensis* strain BMB171, is isolated and characterized together with its endolysin. This phage is the first tadpole-like phage infecting the Bacillus strains. Genomic analysis shows that the phage genome is dissimilar to all those of previously characterized phages, only exhibiting low similarities with partial regions of the *B. thuringiensis* prophages. Phylogenetic analysis revealed that the phage was distant from the other Bacillus phages in terms of evolution. The novel genome sequence, the distant evolutionary relationship, and the special virion morphology together suggest that the phage vB_BthS_BMBphi could be classified as a new phage lineage. The genome of the phage is found to contain a restriction modification system, which might endow the phage with immunity to the restriction modification system of the host bacterium. The function of the endolysin PlyBMB encoded by the phage vB_BthS_BMBphi was analyzed, and the endolysin could lyse all the tested *Bacillus cereus* group strains, suggesting that the endolysin might be used in controlling pathogenic *B. cereus* group strains. The findings of this study enrich the understanding of phage diversity and provide a resource for controlling the *B. cereus* group pathogenic bacteria.

## 1. Introduction

Bacteriophage, the parasite of bacteria, is the most abundant and diverse biological entity in the natural ecosystem [1]. Due to their ability to specifically lyse pathogenic bacteria, phages have been thought of as a novel kind of antimicrobial agent even since their first discovery. Nowadays, due to the generation of antibiotic-resistant pathogenic bacteria, the phage is the subject of renewed attention [2,3]. However, because of the coevolution of phages and their host bacteria, the resistance of the pathogenic bacteria to phages appears at high frequency and the speeds are even faster than the appearance of antibiotic resistance [4]. The construction of phage cocktail using different phages seems to be a promising strategy due to its ability to avoid the generation of bacterial phage-resistance [2,5]. The divergent genetic background of the phages used for constructing phage cocktail is critical for avoiding the generation of phage-resistance, because the diverse phages in the phage cocktail can lyse the pathogenic bacteria by different mechanisms [6]. However, due to the increase in new phage isolates, the newly isolated phages are showing higher and higher similarity with previously isolated phages [7]. Thus, the isolation of phages with novel genetic backgrounds is critical for the application of phage therapy.

*Bacillus thuringiensis* is widely used in producing biological pesticides for its ability to produce insecticidal crystal proteins (ICPs) with controlling activity against problematic insects of the agricultural industry [8]. Although *B. thuringiensis* is not a human pathogen, it is reported to be closely related with some human pathogenic bacteria, including *B. anthracis* and *B. cereus*. These three species, accompanied by *Bacillus weihenstaphanensis*, *Bacillus pseudomycoides*, and *Bacillus cytotoxicus*, are classified as the *Bacillus cereus* group [9]. *B. anthracis* is the pathogen causing human anthrax, and *B. cereus* can cause emesis and diarrhea in humans by producing the emetic toxin and enterotoxins [10,11]. A previous study on phages infecting *B. thuringiensis* revealed that some of the phages could also infect strains of *B. anthracis* and *B. cereus* [12]. Moreover, several of the endolysins, which could also lyse the pathogenic bacteria, produced by *B. thuringiensis* phages were reported to lyse the pathogenic strains of *B. anthracis* and *B. cereus* [13]. Thus, the isolation of new phages infecting *B. thuringiensis* might also provide resources for controlling the other, pathogenic, strains of the *Bacillus cereus* group.

In this study, a novel phage, vB_BthS_BMBphi, infecting the *B. thuringiensis* strain BMB171 has been isolated and characterized. The phage has a tadpole-like virion structure and forms a large plaque with a diameter of about 5 mm on the lawn of strain BMB171. Genomic and phylogenetic analysis of the phage revealed that it is a novel phage and only showed similarities with partial regions of the prophage in the *B. thuringiensis* strain genome. In consideration of the specific virion structure and low genome similarity of the phage with previously characterized phages, it is rational to classify the phage as a novel lineage. The endolysin PlyBMB encoded by the phage vB_BthS_BMBphi could lyse all the tested *B. cereus* group strains, suggesting the endolysin could be used in the control of pathogenic bacteria belonging to the *B. cereus* group.

## 2. Materials and Methods

### 2.1. Bacterial Strains and Growth Conditions

The Bacillus strains used in this work were collected and stored by our lab and used for phage isolation and phage host range determination. The *B. thuringiensis* strain BMB171 is an acrystalliferous mutant that is widely used in constructing genetic engineering strains by expressing insecticidal crystal protein encoding genes applied in the preparation of commercial insecticides [14]. The strains were cultivated in Luria-Bertani (LB) broth medium at 30 °C. Agar at a final concentration of 1.2% or 0.7% was added into the LB broth for the preparation of solid and semisolid media, respectively. Agar at a final concentration of 0.3% was added into the LB broth for preparation of the semisolid medium for testing the mobility of the bacterial strains.

### 2.2. Phage Isolation, Purification, and Observation

The fermentation liquid of the *B. thuringiensis* engineering strain ACE-38 [15], which was constructed by transforming the insecticidal gene into the strain BMB171, was stored at room temperature for 1 year and used for phage isolation. The fermentation liquid was first centrifuged at 12,000× *g* for 10 min and subsequently filtered through a 0.22-μm filter (Millipore, Burlington, MA, USA) to remove the spores and fermentation materials, and then the filtered supernatant was used for phage isolation as previously described using the double-layer overlay method [16]. The phage propagation, the efficiency-of-plating (EOP) test, and the host range determination were all performed by double-layer overlay assay. The storage stability of the phage was also determined by testing the EOP of the phage suspension after storage for different time periods.

To observe the morphology of the phage virion, the phage was purified by using sucrose density gradient centrifugation as previously described [17]. The purified phage suspension was deposited onto cuprum grids with carbon-coated Formvar film, strained with 2% potassium phosphotungstate (pH 7.2), and further air-dried. The sample was observed using a transmission electron microscope (TEM, H-7000FA; Hitachi, Tokyo, Japan) at an acceleration voltage of 100 kV.

### 2.3. Bacterial and Phage Genomic DNA Purification, Genome Sequencing, and Bioinformatic Analysis

The genomic DNA of the phage and the host bacteria were purified according to the methods previously described, respectively [16,18]. The genome of the bacteria and phages were sequenced by using an Illumina Hiseq 2500 (Illumina, San Diego, CA, USA) and assembled into contigs using the software Velvet v1.2.07 [19]. Gaps between contigs of the phage genome were filled by primer walking into complete genome sequences. The proteins in the phage genome were predicted using GeneMarks [20] and the functions of the phage-encoded proteins were predicted by using BLASTP [21]. The genes encoding the putative tRNAs were analyzed using tRNAScan-SE [22]. The functions of the phage proteins were further confirmed by searching against the HHpred database [23] and the Pfam database [24]. The visualization of the phage genome was performed by using Circoletto [25], and comparative genomic analysis of the phage genome was carried out using Gepard [26]. Phylogenetic analysis of the phage and proteins were performed by using MEGA 7.0 [27] with the neighbor-joining method and bootstrap analysis (1000 replicates) of a Clustal alignment, and visualized by using iTOL [28].

### 2.4. Expression and Purification of the Endolysin PlyBMB

The gene *gp*41 was amplified from the phage vB_BthS_BMBphi genome using the primer pairs PlyBMB-For/*Eco*RI (5′-GTGAATTCATGCAAATCAAAACAGATT-3′) and PlyBMB-Rev/*Sal*I (5′-GCGTCGACTTACCCTAAATATTGGAA-3′) and inserted into the vector pET28a to construct the recombinant plasmid pET28a/gp41. The plasmid pET28a/gp41 was transformed into the *Escherichia coli* strain BL-21, and the recombinant strain was used for expression of PlyBMB via induction with isopropyl-B-d-thiogalactopyranoside (IPTG) with a final concentration of 0.4 mM. The protein was further purified using nickel nitrilotriacetic acid chromatography (Ni-NTA columns; Qiagen, Dusseldorf, Germany) and buffer-exchanged into 50 mM Tris, 300 mM NaCl, 10% glycerol, and 1 mM β-mercaptoethanol, pH 8.0, and stored at −20 °C until further use.

### 2.5. Lytic Activity Assay of PlyBMB

To analyze the influence of protein concentration on the lytic activity, 200 μL of exponential growth phase *B. thuringiensis* strain BMB171 in LB broth was collected by centrifugation at 8000× *g* for 1 min, and the pellets were washed twice using 50 mM Tris, 300 mM NaCl, and 10% glycerol and resuspended in 50 mM Tris, 300 mM NaCl, and 10% glycerol to the final absorbance value at 600 nm of 0.8. The protein PlyBMB was added into the bacterial suspension to different final concentrations. For the purpose of testing the optimal lytic pH, the exponential growth phase BMB171 was resuspended in buffers of different pH, including pH 1.0 and 2.0 (50 mM Na_2_HPO_4_–Cl, 300 mM NaCl, and 10% glycerol); pH 3.0, 4.0, 5.0, and 6.0 (50 mM Na2HPO4–citric acid, 300 mM NaCl, and 10% glycerol); pH 7.0, 8.0, 9.0, and 10.0 (50 mM Tris–Cl, 300 mM NaCl, and 10% glycerol); and pH 11.0 and 12.0 (50 mM Na2CO3–NaHCO3, 300 mM NaCl, and 10% glycerol), and the purified PlyBMB protein was added into the bacterial suspension to a final concentration of 2 μM. The lytic spectrum of the endolysin PlyBMB was determined using the method used for lytic activity assay. For lytic activity assay of Gram-negative strains, EDTA was added into the reaction mixture to a final concentration of 50 mM to increase the permeability of the bacterial outer membrane [29,30]. The changes in bacterial concentration were determined by detecting the turbidity of the bacterial suspension at 600 nm.

### 2.6. GenBank Accession Number

The genome of the phage vB_BthS_BMBphi was deposited in the GenBank database under the accession number MH458951.

## 3. Results

### 3.1. Phage vB_BthS_BMBphi Exhibits High Lytic Activity and High Specificity

**Figure 1:**
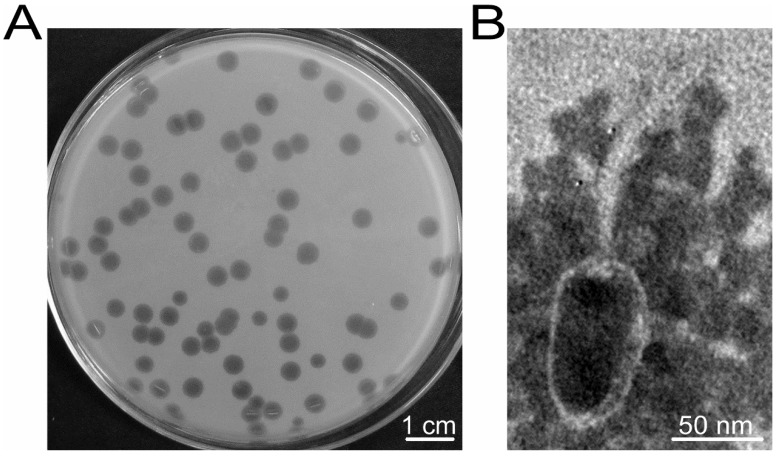
Morphologies of the plaques (**A**) and virion particle (**B**) of the phage vB_BthS_BMBphi.

As a widely used recipient strain for constructing genetically engineered strains, no phages infecting BMB171 have been reported. In this study, using the fermentation agent produced by ACE-38 as the sample and using BMB171 as the indicator strain, one phage, designated as vB_BthS_BMBphi, which formed a large clear plaque of approximately 5 mm in diameter, was isolated (Figure 1A). The phage exhibited high lytic activity against the host strain BMB171 and could lyse the strain in the exponential growth phase at a multiplicity of infection (MOI) of lower than 0.001. Host range analysis of the phage vB_BthS_BMBphi revealed that the phage is highly specific and only infected the *B. thuringiensis* strain BMB171 among the 62 tested strains, including 54 *B. thuringiensis* strains of 53 different serotypes and strains of *B. cereus*, *B. anthracis*, and some other Gram-negative species (Table S1).

The phage virion was observed by TEM and the result showed that the phage was a Siphoviridae family phage that has a tadpole-like virion particle (Figure 1B). The head of the phage has an oval shape with a length of 93 nm and a widest width of 52 nm. The phage has a long, noncontractile tail of approximately 128 nm. According to previous reports, the Siphoviridae family phages infecting Bacillus strains mainly contain an icosahedral head, and, to our knowledge, no Bacillus phage with an oval head had been isolated until now, suggesting that the phage might represent a new type of Bacillus phage.

### 3.2. General Genomic Characteristics of the Phage vB_BthS_BMBphi

**Figure 2:**
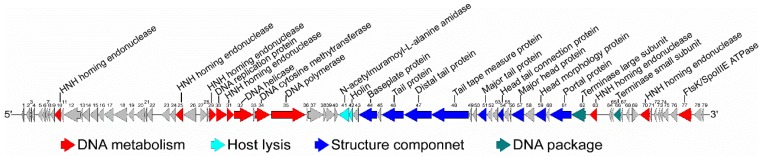
Schematic diagram of the genome of the phage vB_BthS_BMBphi. The functional classification of the genes in the phage vB_BthS_BMBphi genome are indicated in different colors and the transcription directions of the genes are also shown.

Due to the potential novelty of the phage vB_BthS_BMBphi, the genome of the phage was sequenced and analyzed. The phage has a linear genome of 49,277 bp in length, and the G + C content of the phage genome is 39.71%, which is higher than that of the host strain BMB171 (the G + C content of the BMB171 chromosome is 35.3%) [14]. In total, 79 putative coding sequences (CDSs) with an average length of 547.5 bp were predicted to be encoded by the phage genome, and the average distance between consecutive CDSs was 75.3 bp (Table S2). No tRNA was found in the phage genome by searching the genome with tRNAScan-SE. A comprehensive search of the NR database for homologs of the 79 CDSs returned 53 significant matches (*E* value ≤ 10^−3^), while the other 26 CDSs were specific for the phage vB_BthS_BMBphi. Among the 53 matched CDSs, 27 of the CDSs exhibited similarity with other phage proteins, including proteins from Bacillus phages and phages of some other bacterial species, such as phages infecting strains of Enterococcus, Streptococcus, Clostridium, and Listeria. The genome of the phage vB_BthS_BMBphi shows a modular structure and genes with associated function are clustered (Figure 2). The proteins encoded by the phage genome are mainly associated with the functions of phage genome replication, phage particle composition, and host lysis.

The majority of the phage genes are transcribed in reverse, except for 15 genes which are transcribed in the forward direction, of which 14 are clustered in the phage genome; these 14 genes are mainly responsible for phage genome replication, including a DNA replication protein, DNA helicase, and DNA polymerase. One interesting finding was that one DNA cytosine methyltransferase (Gp34), which is the main component of the bacterial restriction modification (RM) system, was found to be located in this region. Another gene (*gp*31), which was annotated as an HNH endonuclease, was found to be located upstream of the putative DNA cytosine methyltransferase. Bacteria employ defensive strategies to avoid phage infection, such as the restriction modification (RM) system, which can recognize specific sites on the foreign DNA and cleave the nonmodified phage genome [31,32]. Function prediction of the proteins Gp31 and Gp34 was conducted using REBASE, and the result showed that Gp31 and Gp34, respectively, had similarity with BceB24ORF5584P, which is a putative type II restriction enzyme of unknown recognition sequence that could cleave DNA, and M.BceB24ORF5584P, which was annotated as a putative type II cytosine-5 DNA methyltransferase of unknown recognition sequence that could catalyze the methylation of DNA, suggesting these proteins belong to the type II RM system [33]. BceB24ORF5584P was located immediately upstream of protein M.BceB24ORF5584P in the *Bacillus mycoides* BtB2–4 genome. The DNA helicase (Gp32) between GP31 and Gp34 might have been obtained by genome recombination. The host strain BMB171 also contained the type II RM system. The type II RM system harbored in the phage genome might catalyze the methylation of the phage genome and endow the phage with resistance to the host RM system. Besides the classical restriction modification system, the host strain BMB171 also had the methylation-dependent (type IIM) restriction endonuclease Bth171I (specificity: Gm5CNGC), which cleaves only modified DNA and is inactive on unmodified DNA [14,33]. Further analysis of the specificity of the RM system encoded by the phage vB_BthS_BMBphi will establish the possible influence of the restriction endonuclease Bth171I on the infection of the phage.

Besides the genes associated with the phage genome replication, six HNH endonucleases (Gp10, Gp25, Gp29, Gp31, Gp63, and Gp70), which are associated with nucleotide metabolism, were found to be encoded by the phage genome. These six HNH endonucleases exhibit diverse amino acid sequences and are scattered throughout the phage genome (Figure S1). The HNH endonuclease was reported to be responsible for the mobility of the phage genes and might cause the mosaic genome structure of phages by cleaving the phage genome at specific recognition sites [34,35]. The HNH endonuclease-encoding genes *gp*10 and *gp*25 are located far from any of the functional genomic modules in the phage genome, and the functions of these two genes need to be further analyzed. The gene *gp*30, which is between the two endonuclease-encoding genes *gp*29 and *gp*31, was annotated as encoding a DNA replication protein and shows highest similarity with the genes from the *B. thuringiensis*. The close proximity of the DNA replication protein and endonuclease gene locations in the phage genome suggest that the DNA replication protein of the phage might have been transferred from the genome of the *B. thuringiensis* strain. On the other hand, the phage genome’s metabolism-associated gene cluster is transcribed in a different direction to the other genes in the phage genome, and different genes in this module exhibited highest similarity with genes of different phages. The result indicated that the phage genome’s metabolism-associated genes might be obtained from different phages in whole or in part. According to previous reports, the endonuclease from the phage T4 formed a so-called packsome accompanied by the phage terminase and was responsible for the packaging of the phage genome [34,36]. The other two endonucleases, Gp63 and Gp70, which are located near the terminase in the phage vB_BthS_BMBphi genome, might take part in the packaging of the phage genome.

### 3.3. The Phage vB_BthS_BMBphi Contains a Novel Genome

**Figure 3:**
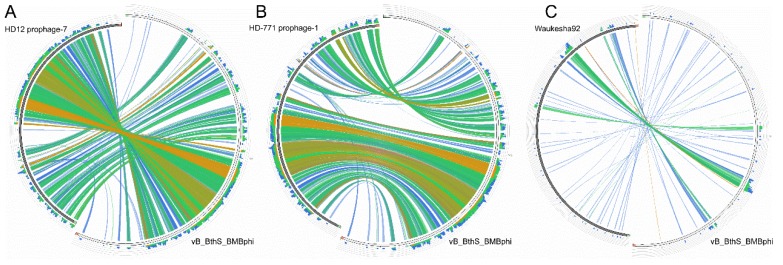
Comparative genomic analysis of phage vB_BthS_BMBphi. The genome sequences of phage vB_BthS_BMBphi, HD12 prophage-7 (**A**), HD-771 prophage-1 (**B**), and phage Waukesha92 (**C**), were compared and visualized by using Circoletto, and genome regions that exhibited similarity are shown in the same color. The degrees of similarities are also shown.

**Figure 4:**
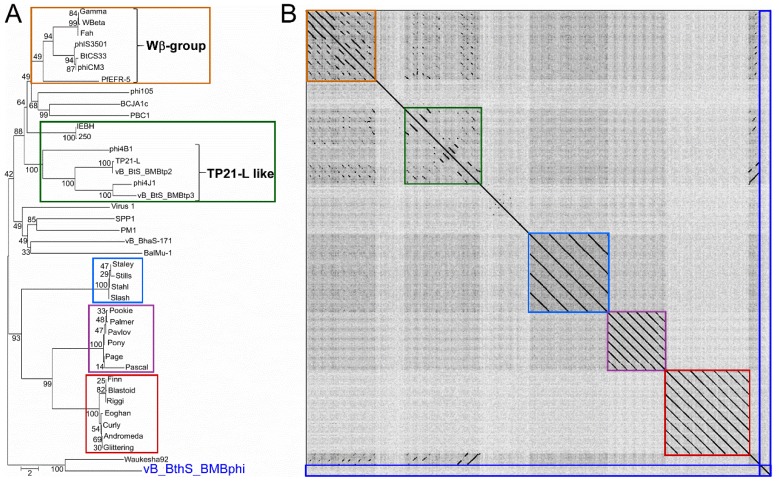
Phylogenetic and comparative analysis of vB_BthS_BMBphi. (**A**) Phylogenetic analysis of the Siphoviridae family phages infecting the Bacillus species. The genome sequences of 40 Siphoviridae family Bacillus phages were collected from GenBank and used for phylogenetic tree analysis together with the genome of the phage vB_BthS_BMBphi. (**B**) Comparative genomic analysis of vB_BthS_BMBphi. The genomes used for comparative genomic analysis were listed as in the order shown in Figure 4A. The information of the Bacillus phages used for phylogenetic and comparative genomic analysis is shown in Table S3.

The genome of the phage vB_BthS_BMBphi was searched against the NR database in National Center for Biotechnology Information (NCBI) to find similar phages, and the result showed that only partial regions of the vB_BthS_BMBphi genome showed similarity with chromosome regions of the *B. thuringiensis* strains HD-771 (GenBank accession number NC_018500.1) and HD12 (GenBank accession number NC_CP014847.1), but not the other phage genomes. These two bacterial genome regions were predicted to be intact prophages by using PHAST [37], which indicated that the phage might be derived from the prophages of *B. thuringiensis* (Figure S2). Furthermore, the regions that exhibited similarity with the prophage sequences were located in the middle of the phage genome, while no homologous genomic regions were found to have similarity with the terminus of the vB_BthS_BMBphi genome, indicating that the phage has undergone genome recombination during the evolutionary process. The genes in the structure module of the phage genome showed higher similarities with genes in the bacterial genome than the genes in the DNA metabolism module. Although the DNA metabolism genome module and the structural module in the phage vB_BthS_BMBphi genome showed a collinearity relationship with the prophages, the whole genome of the phage vB_BthS_BMBphi exhibited a rearranged genome structure compared to that of the prophage, with the reversion of the two modules, which might have occurred during the lytic conversion of the prophage or the lysogeny conversion of the phage (Figure 3). Phylogenetic analysis of the Siphoviridae family phages infecting Bacillus genus strains revealed that phage vB_BthS_BMBphi was only close with Waukesha92 in terms of evolution and distant in evolution to the other Bacillus phages (Figure 4A). Comparative genomic analysis of the phages vB_BthS_BMBphi and Waukesha92 revealed that although these two phages showed a close relationship in the phylogenetic tree, they only maintained a few similar genome regions (Figure 3C). The integral similarity between these two phages was analyzed by using BLASTn and the result showed that they only showed extremely low similarity. The pairwise proteomic comparation of these Bacillus phages also showed that there was no collinearity relationship between the phage vB_BthS_BMBphi and the other Bacillus phages (Figure S3). The comparative genomic and proteomic analysis of the phage vB_BthS_BMBphi indicates that the phage vB_BthS_BMBphi might be representative of a novel phage lineage.

### 3.4. The Phage vB_BthS_BMBphi Genome Contains a Repeated Terminal Structure

**Figure 5:**
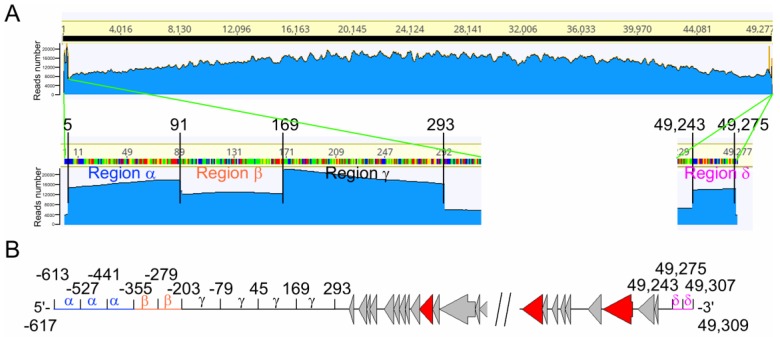
Terminal structure analysis of the phage vB_BthS_BMBphi genome. (**A**) Assembly of the phage genome. Using the assembled phage genome as the template, the reads archived by genome sequencing were mapped onto the phage genome. The number of the reads that were mapped to the phage genome is shown. (**B**) Predicted terminal genome structure of the phage vB_BthS_BMBphi. The repeat numbers of the repeated regions are indicated.

The phages might harbor a repeated genome terminal structure to protect their genomes [38]. For the purpose of identifying the terminus of the phage genome, the reads acquired by genome sequencing were mapped onto the phage genome by using Geneious R11. The result showed that the reads that were mapped to the phage genome termini were more than those mapped to the nearby regions (Figure 5A). The 5′-terminus of the phage vB_BthS_BMBphi genome, which can be divided into three regions, contained two more repeats of region α (120 bp-length), one more repeat of region β (77 bp-length), and three more repeats of region γ (125 bp-length), indicating that the terminus of the phage genome might contain, in total, three repeats of region α, two repeats of region β, and four repeats of region γ (Figure 5B). At the 3′-terminus of the phage genome, one more repeat of region σ was found, suggesting the 3′-terminus of the phage genome contain two repeats of region σ, in total. In consideration of the repeated regions in the phage genome termini, the total length of the phage genome might be 49,921 bp. Functional analysis of the repeated regions showed that these regions do not contain functional protein-encoding genes. The repeated regions were also confirmed by primer-walking and Sanger sequencing and the result was identical with respect to the repeat number of the terminal repeated regions.

### 3.5. Lytic Activity of the Endolysin PlyBMB

**Figure 6:**
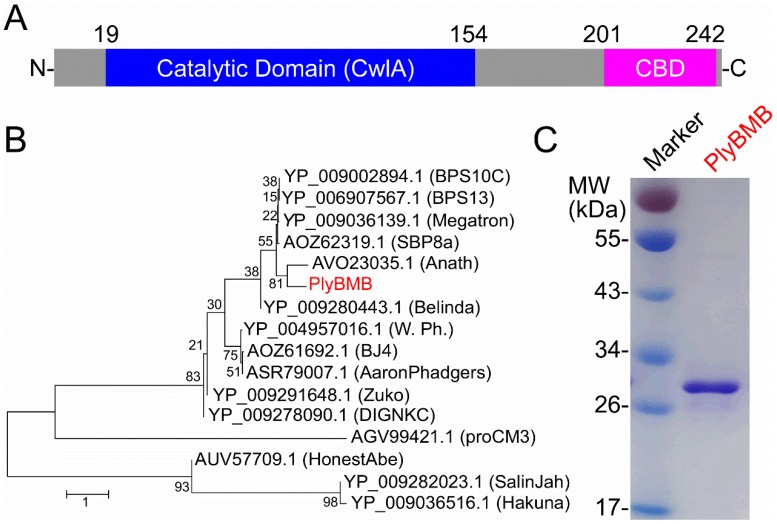
Bioinformatic analysis and expression of PlyBMB. (**A**) Domain composition of PlyBMB. The locations of the catalytic domain and the cell wall-binding domain (CBD) are shown. (**B**) Phylogenetic analysis of PlyBMB with the other endolysins encoded by Bacillus phages. Only the endolysins showing more than 50% similarity with PlyBMB were used for phylogenetic analysis. The GenBank accession number of the endolysins and the names of the phages that encoded the endolysins are shown. (**C**) Expression and purification of PlyBMB.

The endolysin exhibits high potential for being used in the control of pathogenic bacteria, due to its ability to lyse the bacterial cell wall [39,40,41]. Bioinformatic analysis of the phage vB_BthS_BMBphi genome revealed that the gene *gp*41 was annotated to encode an endolysin and *gp*42 was annotated to encode a holin, which can facilitate the access of endolysin to the cell wall by forming holes in the cell membrane [42]. The phage vB_BthS_BMBphi isolated in this study showed high lytic activity against the host bacteria, suggesting the phage might encode an endolysin with high lytic activity. The bioinformatic analysis of the endolysin (GP41, designated as PlyBMB) showed that the protein contained a N-terminal catalytic domain and a C-terminal cell wall-binding domain (Figure 6A). The Bacillus phages encoding endolysins that exhibited more than 50% similarity with PlyBMB was used for constructing a phylogenetic tree. The result showed that the function of all the endolysins used for phylogenetic tree analysis have not been analyzed, indicating that the endolysin analyzed in this study was a novel endolysin (Figure 6B). The alignment of the amino acid sequences of the endolysins used for phylogenetic analysis showed that all these endolysins showed highly conserved sequences, with four residues conserved in all the analyzed endolysins, which might be essential for the function of the endolysins (Figure S4). The endolysin encoded by the Bacillus phage Anath (GenBank accession number MG983742.1) showed the closest relationship with PlyBMB in terms of evolution, while the phage vB_BthS_BMBphi exhibited no similarity with the phage Anath. The endolysin PlyBMB has a molecular weight of 27 kDa and is easily prepared in a soluble form (Figure 6C).

**Figure 7:**
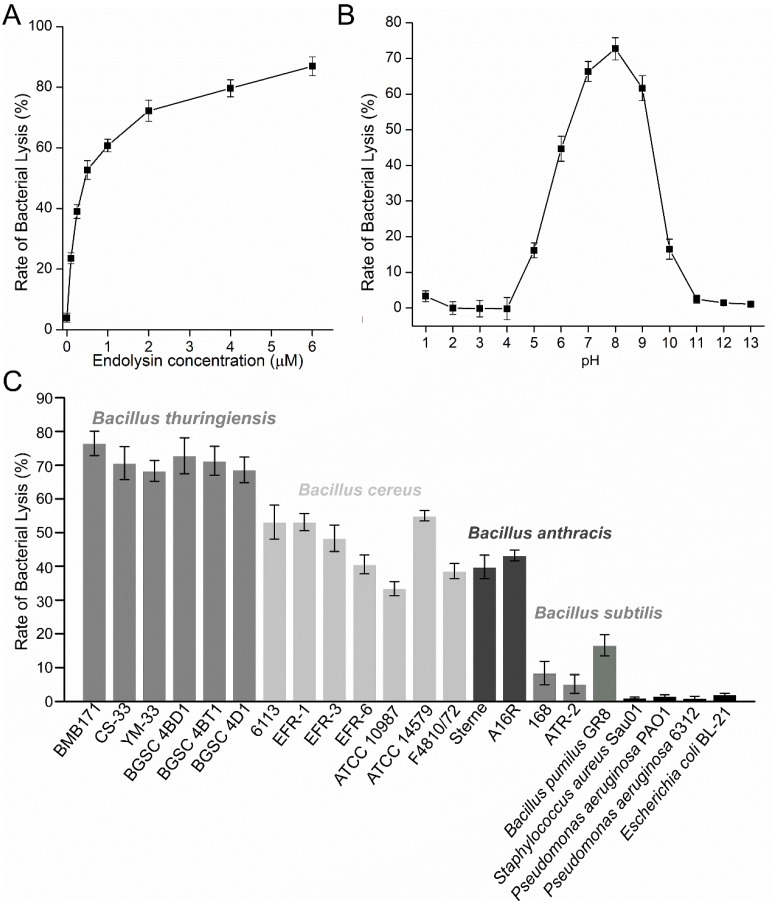
Lytic activity analysis of the endolysin PlyBMB. (**A**) The influence of concentration on the lytic activity of PlyBMB. (**B**) The influence of pH on the lytic activity of PlyBMB. The *B. thuringiensis* strain BMB171 was used for the optimal pH test. (**C**) Lytic spectrum of PlyBMB.

The lytic activity of the endolysin PlyBMB was determined and the result showed that the endolysin could lyse more than 87% of the cells at a concentration of 6 μM PlyBMB (Figure 7A). At a concentration of 2 μM, PlyBMB exhibited a relatively high lytic activity and could lyse more than 72% of the cells, and thus the concentration of 2 μM was used for the following tests. The optimal lytic pH test showed that PlyBMB exhibited lytic activities between pH 5.0 and pH 10.0, with the highest lytic activity at pH 8.0 (Figure 7B). Lytic spectrum analysis showed that the endolysin PlyBMB could lyse all the tested *B. cereus* group strains, including six tested *B. thuringiensis* strains, seven tested *B. cereus* strains, and two *B. anthracis* strains (Figure 7C). The endolysin PlyBMB also exhibited low lytic activity with the tested *B. pumilus* and *B. subtilis* strains, but not the tested Gram-negative strains. The finding suggested that PlyBMB was relatively specific against the *B. cereus* group strains and could be used in the biocontrol of the pathogenic *B. cereus* group strains.

## 4. Discussion

As the most abundant biological entities in earth’s ecosystems, phages are predicted to be highly diverse, which provides much potential for the development of phage therapies [43,44]. However, along with the increase in the number of new phage isolates, the newly isolated phages exhibit higher and higher similarity with previously characterized phages, suggesting that we might have overestimated the diversity of the phage [7]. As shown in this study, 31 of the 41 Siphoviridae family Bacillus phages could be clustered into different clusters. Nevertheless, the isolation of phages with novel genetic backgrounds is critical for the development of phage therapies. In this study, the isolation of a novel phage, vB_BthS_BMBphi, infecting *B. thuringiensis*, has been presented. The phage possesses a novel genome sequence and shows no substantial similarity to any of the available previously isolated phages. This is also the first time that the special tadpole-like virion structure has been found in a Bacillus phage. Comparative genomic and phylogenetic analysis showed that the phage is evolutionarily distant from the other available Siphoviridae family Bacillus phages. In consideration of all the above results, it is rational to classify the phage as belonging to a novel phage lineage.

*B. thuringiensis* is widely used in the production of pesticides for its ability to produce insecticide crystal proteins (Cry proteins) [8]. Phage contamination during *B. thuringiensis* fermentation was found to severely harm the yield of insecticide crystal proteins [45]. The strain BMB171 is widely used as a recipient bacterium in the construction of *B. thuringiensis* engineering strains for producing biological insecticides, and to date, there is no phage that has been found to infect the strain BMB171. The phage vB_BthS_BMBphi analyzed in this study was isolated from the fermentation liquid of the strain BMB171 and showed high lytic activity against the strain BMB171. The finding of this study indicated that the use of BMB171 as the recipient bacterium for constructing *B. thuringiensis* engineering strains might lead to phage contamination during the fermentation of the engineering strain. Thus, the further construction of phage-resistant *B. thuringiensis* recipient strains will be necessary.

Similarly to phages themselves, endolysins encoded by phages have also been thought of as a novel kind of antimicrobial substance for their ability to lyse bacterial cell walls from the outside [41,46]. According to previous reports, the endolysins usually exhibit a broader lytic spectrum than the producing phage [47]. As shown in this study, the endolysin PlyBMB encoded by the phage vB_BthS_BMBphi exhibited a broader lytic spectrum than the phage vB_BthS_BMBphi. The potential reason might be due to the fact that each process of the phage life cycle, including the adsorption, injection of genomic DNA, phage virion replication, production of endolysin, transport of the endolysin to the cell wall, and finding an available catalytic site of the endolysin in the cell wall, was essential for achieving effective lysis of the bacterium by the phage from within. On the contrary, the endolysin lysing the bacterium from the outside is only determined by the accessibility of the endolysin to the cell wall and the availability of the catalytic site of the endolysin in the cell wall. The endolysin also showed a faster lytic speed than the phage due to its direct lytic activity on the bacterial cell wall. The endolysin analyzed in this study, PlyBMB, is a novel endolysin, and the functions of the endolysins which showed similarity with PlyBMB have not yet been analyzed. The protein showed a broad lytic spectrum against all the tested *B. cereus* group strains, suggesting that the endolysin could be used in the control of pathogenic *B. cereus* group strains.

## Supplementary Materials

The following are available online at http://www.mdpi.com/1999-4915/10/11/611/s1. Table S1: Host range of phage vB_BthS_BMBphi (indicated as BMBphi) and the mutant phage vB_BthS_BMBphi-M (indicated as BMBphi-M), Table S2: General features of the predicted proteins encoded by phage vB_BthS_BMBphi genome, Table S3: Information of phage genomes used in this study, Figure S1: Alignment of the HNH endonucleases encoded by phage vB_BthS_BMBphi, Figure S2: BLASTN analysis of the genome sequence of phage vB_BthS_BMBphi. Figure S3: Comparative proteome analysis of vB_BthS_BMBphi. Figure S4: Amino acid sequence alignment of PlyBMB with the other endolysins encoded by Bacillus phages.
