# Supplementary material for: Isolation of A Novel Bacillus thuringiensis Phage Representing A New Phage Lineage and Characterization of Its Endolysin
Source: Viruses. 2018 Nov 6;10(11):611. doi: 10.3390/v10110611 (PMC6266608; doi:10.3390/v10110611)
Supplement: Supplementary file 1 [file viruses-10-00611-s001.pdf]

Article

Isolation of A Novel *Bacillus Thuringiensis* Phage Representing A New Phage Lineage and Characterization of Its Endolysin

Yihui Yuan [1,2], Qin Peng [3], Shuo Yang [1], Shaowen Zhang [1], Yajuan Fu [2], Yan Wu [2], Meiying Gao [2,*]

1 State Key Laboratory of Marine Resource Utilization in South China Sea, Hainan University, Haikou 570228, P. R. China; yuanyh@hainu.edu.cn (Y.Y.); q50608868@qq.com (S.Y.); 1287070341@qq.com (S.Z.)

2 Wuhan Institute of Virology, Chinese Academy of Sciences, Wuhan 430071, P. R. China; fuyajuann@163.com (Y.F.); wuyan_81@126.com (Y.W.)

3 Ministry of Education Key Laboratory for Ecology of Tropical Islands, College of Life Sciences, Hainan Normal University, Haikou 571158, P. R. China; pengqin1019@126.com (Q.P.)

* Correspondence: mygao@wh.iov.cn

Table S1. Host range of phage vB_BthS_BMBphi (indicated as BMBphi) and the mutant phage vB_BthS_BMBphi-M (indicated as BMBphi-M).

Bacillus thuringiensis									
**Serotypes**	**Serovar**	**Strain**	**Sensitivity**		**Serotypes**	**Serovar**	**Strain**	**Sensitivity**	
			**BMBphi**	**BMBphi-M**				**BMBphi**	**BMBphi-M**
8a, 8b[a]	*kurstaki*	BMB171	+[b]	+[b]	3a, 3b, 3c	*kurstaki*	CS33	-	-
2	*finitimus*	HD-3	-	-	38	*oswaldocruzi*	T38001	-	-
3a, 3c	*alesti*	HD-4	-	-	40	*huazhongensis*		-	-
3a, 3b, 3c	*kurstaki*	HD-73	-	-	41	*sooncheon*	T41001	-	-
4a, 4b	*sotto*	HD-930	-	-	42	*jinghongiensis*	YGd22-03	-	-
4a, 4c	*kenyae*	HD-5	-	-	45	*roskildiensis*	T45001	-	-
5a, 5c	*canadensis*	HD-554	-	-	46	*chanpaisis*		-	-
7	*aizawai*	HD-11	-	-	47	*wratislaviensis*	PO12	-	-
8a, 8b	*morrisoni*	HD-12	-	-	49	*muju*	A39	-	-
8a, 8c	*ostriniae*	HD-501	-	-	51	*xiaguangiensis*	3397	-	-
9	*tolworthi*	HD-537	-	-	53	*asturiensis*	EA34594	-	-
10a, 10b	*darmstadiensis*	HD-146	-	-	54	*poloniensis*	Pbt23	-	-
11a, 11b	*toumanoffi*	HD-201	-	-	55	*palmanyolensis*	EA40694	-	-
11a, 11c	*kyushuensis*	HD-541	-	-	56	*rongseni*	Scg04-02	-	-
12	*thompsoni*	HD-542	-	-	57	*pirenaica*	NA210	-	-
14	*israelensis*	HD-567	-	-	58	*argentinensis*	A20	-	-
16	*indiana*	HD-521	-	-	59	*iberica*	L60	-	-
17	*tohokuensis*	HD-866	-	-	60	*pingluonsis*	NXP15-04	-	-
19	*tochigiensis*	HD-868	-	-	61	*sylvestriensis*	Pbt53	-	-
20a, 20b	*yunnanensis*	HD-977	-	-	62	*zhaodongensis*	HZ39-04	-	-
20a, 20c	*pondicheriensis*	HD-1011	-	-	63	*bolivia*	T63001	-	-
21	*colmeri*	HD-847	-	-	65	*pulsiensis*		-	-

22	*shandongiensis*	HD-1012	-	-	66	*graciosensis*		-	-
31	*toguchini*	*toguchini*	-	-	67	*vazensis*	Ea14696	-	-
35	*seoulensis*	T35001	-	-	68	*thailandensis*	T68001	-	-
36	*malaysiensis*	T36001	-	-	69	*pahangi*	T69001	-	-
37	*andaluciensis*	T37001	-	-	70	*sinensis*	YK30-04	-	-

Other strains

Strains	Sensitivity		Strains	Sensitivity	
	BMBphi	BMBphi-M		BMBphi	BMBphi-M
B. anthracis 63002	-	-	*B. anthracis* 63605	-	-
B. cereus F4810/72			*B. cereus* ATCC10087		
B. pumilius GR8	-	-	*B. subtilis 168*	-	-
Staphylococcus aureus Sau01	-	-	*Escherichia coli* BL-21	-	-
Pseudomonas aerugonisa PAO1	-	-	*Yersinia pseudotuberculosis YPIII*	-	-

[a]The *B. thuringiensis* strain used in this study were mainly the reference strains, except strain BMB171 and CS-33; [b] "+" indicated the strain was sensitive to tested phage and "-" indicated the strain was not sensitive to tested phages.

Table S2. General features of the predicted proteins encoded by phage vB_BthS_BMBphi genome.

ORF	Strand	Left End	Right End	Size (aa)	Closest hit (e value)	The best match in phages (e value)	Predictive Function
ORF01	-	758	904	48			hypothetical protein
ORF02	-	1076	1336	86	*Bacillus thuringiensis* (2e-04)		hypothetical protein
ORF03	-	1338	1448	36			hypothetical protein
ORF04	-	1445	1669	74	*Bacillus thuringiensis* (2e-11)		hypothetical protein
ORF05	-	1913	2218	101	*Bacillus thuringiensis* (3e-35)	*Bacillus* phage Claudi (4e-05)	hypothetical protein
ORF06	-	2220	2405	61	*Bacillus thuringiensis* (6e-14)		hypothetical protein
ORF07	-	2407	2604	65			hypothetical protein
ORF08	-	2604	2738	44			hypothetical protein

ORF09	-	2776	3078	100	*Bacillus cereus* (7e-11)		hypothetical protein
ORF10	-	3068	3514	148	*Streptococcus pneumoniae* (5e-38)	*Bacillus* phage PBC6 (5e-32)	HNH homing endonuclease
ORF11	-	3504	3728	74			hypothetical protein
ORF12	-	3730	4911	393	*Bacillus thuringiensis* (2e-100)		hypothetical protein
ORF13	-	4908	5045	45			hypothetical protein
ORF14	-	5042	5539	165			hypothetical protein
ORF15	-	5563	6123	186	*Bacillus cereus* (6e-53)	*Bacillus* phage phiAGATE (1e-17)	hypothetical protein
ORF16	-	6123	6566	147	*Bacillus cereus* (7e-17)		hypothetical protein
ORF17	-	6569	7213	214			hypothetical protein
ORF18	-	7246	8193	315	*Bacillus thuringiensis* (2e-95)	*Enterococcus* phage vB_EfaS_IME198 (7e-61)	DNA primase
ORF19	-	8270	8674	134			hypothetical protein
ORF20	-	8791	9402	203			hypothetical protein
ORF21	-	9404	9583	59			hypothetical protein
ORF22	-	9585	9956	123	*Bacillus* phage MG-B1 (4e-17)	*Bacillus* phage MG-B1 (4e-17)	hypothetical protein
ORF23	-	10616	11173	185	*Bacillus thuringiensis* (6e-30)		hypothetical protein
ORF24	-	11238	11594	118			hypothetical protein
ORF25	-	11607	12134	175	*Bacillus thuringiensis* (2e-77)	*Bacillus* phage PBC2 (4e-68)	HNH endonuclease
ORF26	-	12147	13037	296	*Bacillus thuringiensis* (6e-46)		hypothetical protein
ORF27	+	13343	13825	160	*Bacillus thuringiensis* (8e-41)		hypothetical protein
ORF28	+	13826	14008	60			hypothetical protein
ORF29	+	13986	14483	165	*Bacillus* phage Mater (6e-30)	*Bacillus* phage Mater (6e-30)	HNH homing endonuclease
ORF30	+	14480	15238	252	*Bacillus thuringiensis* (3e-118)		DNA replication protein
ORF31	+	15238	15723	161	*Bacillus* phage Bobb (1e-25)	*Bacillus* phage Bobb (1e-25)	HNH homing endonuclease
ORF32	+	15725	17095	456	*Bacillus thuringiensis* (3e-170)	*Streptococcus* phage SPQS1 (9e-110)	DNA helicase
ORF33	+	17085	17252	55			hypothetical protein
ORF34	+	17245	18267	340	*Streptococcus pneumoniae* (5e-161)	*Bacillus* phage PK16 (6e-104)	DNA cytosine methyltransferase
ORF35	+	18378	20774	798	*Bacillus thuringiensis* (0.0)	*Listeria* phage LP-037 (2e-127)	DNA polymerase
ORF36	+	20854	20958	34			hypothetical protein
ORF37	+	20951	22057	368	*Bacillus thuringiensis* (4e-105)	*Paenibacillus* phage Tripp (9e-46)	hypothetical protein

ORF38	+	22054	22236	60			hypothetical protein
ORF39	+	22238	22846	202	*Bacillus thuringiensis* (1e-82)	*Enterococcus* phage SAP6 (3e-09)	hypothetical protein
ORF40	+	22847	23029	60			hypothetical protein
ORF41	-	23113	23847	244	*Bacillus cereus* (7e-105)	*Bacillus* phage BJ4 (7e-89)	N-acetylmuramoyl-L-alanine amidase
ORF42	-	23847	24095	82	*Bacillus thuringiensis* (6e-34)		Holin
ORF43	-	24108	24506	132	*Bacillus thuringiensis* (3e-17)		hypothetical protein
ORF44	-	24522	25805	427	*Bacillus thuringiensis* (5e-90)	*Bacillus thuringiensis* (0.0)	Receptor binding baseplate protein
ORF45	-	25820	26098	92	*Bacillus thuringiensis* (5e-42)		hypothetical protein
ORF46	-	26136	27692	518	*Bacillus thuringiensis* (0.0)		Tail protein
ORF47	-	27725	29656	643	*Bacillus thuringiensis* (0.0)		Distal tail protein
ORF48	-	29637	32258	873	*Bacillus thuringiensis* (0.0)	*Bacillus* phage phi4B1 (2e-63)	Tail tape measeure protein
ORF49	-	32294	32461	55	*Bacillus thuringiensis* HD-771 (1e-23)		hypothetical protein
ORF50	-	32554	32850	98	*Bacillus thuringiensis* (3e-49)		hypothetical protein
ORF51	-	32913	33518	201	*Bacillus thuringiensis* (2e-124)		Major tail protein
ORF52	-	33544	33945	133	*Bacillus thuringiensis* (4e-74)		hypothetical protein
ORF53	-	33950	34342	130	*Bacillus thuringiensis* (1e-68)		hypothetical protein
ORF54	-	34329	34712	127	*Bacillus thuringiensis* (1e-65)		Head tail connection protein
ORF55	-	34713	35117	134	*Bacillus thuringiensis* (2e-60)		hypothetical protein
ORF56	-	35151	35252	33			hypothetical protein
ORF57	-	35279	36160	293	*Bacillus cereus* (1e-112)	*Enterococcus* phage SAP6 (8e-44)	Major head protein
ORF58	-	36229	36873	214	*Bacillus cereus* (6e-71)	*Clostridium* phage phi8074-B1 (3e-25)	hypothetical protein
ORF59	-	36929	37702	257	*Bacillus thuringiensis* (4e-82)		Phage head morphologies protein
ORF60	-	37724	37984	86	*Bacillus cereus* (3e-46)	*Bacillus* phage JL (8e-34)	hypothetical protein
ORF61	-	37985	39517	510	*Bacillus cereus* (0.0)	*Clostridium* phage phi8074-B1 (1e-83)	Portal protein
ORF62	-	39517	40524	335	*Bacillus cereus* (8e-180)	*Clostridium* phage phi8074-B1 (5e-138)	Terminase large subunit
ORF63	-	40806	41312	168	*Bacillus eiseniae* (6e-26)	*Bacillus* phage Stahl (5e-20)	HNH endonuclease
ORF64	-	42176	42346	56			hypothetical protein
ORF65	-	42343	42483	46			hypothetical protein
ORF66	-	42480	42977	165	*Bacillus thuringiensis* (2e-48)	*Enterococcus* phage VD13 (2e-14)	Terminase small subunit

ORF67	-	42990	43169	59			hypothetical protein
ORF68	-	43376	43606	76			hypothetical protein
ORF69	+	43796	44275	159	*Bacillus thuringiensis* (4e-14)		hypothetical protein
ORF70	-	44356	45024	222	*Bacillus thuringiensis* (1e-74)	*Bacillus* phage PBC6 (9e-23)	HNH endonuclease
ORF71	-	45021	45182	53			hypothetical protein
ORF72	-	45337	45531	64			hypothetical protein
ORF73	-	45615	45773	52			hypothetical protein
ORF74	-	45767	45952	61	*Streptococcus pneumoniae* (8e-11)		hypothetical protein
ORF75	-	45942	46301	119	*Bacillus thuringiensis* (8e-25)		hypothetical protein
ORF76	-	46521	46958	145	*Bacillus cereus* (5e-54)	*Bacillus* phage Leo2 (3e-34)	hypothetical protein
ORF77	-	47020	47985	321	*Streptococcus pneumoniae* (9e-27)	*Bacillus* phage Curly (2e-97)	FtsK/SpoIIIE ATPase
ORF78	-	48175	48699	174	*Salmonella enterica* (2e-39)	*Bacillus* phage Mater (9e-32)	hypothetical protein
ORF79	-	48704	48832	42			hypothetical protein

Table S3. Informations of phage genomes used in this study.

Phage	GenBank Accession No.	Genome Length (bp)
Bacillus virus 1	NC_009737	35055
Bacillus virus BMBtp2	NC_019912	36932
Bacillus phage Gamma	NC_007458	37253
Bacillus phage TP21-L	NC_011645	37456
Bacillus phage Fah	NC_007814	37974
Bacillus phage phi4B1	NC_028886	38663
Bacillus phage phiCM3	NC_023599	38772
Bacillus phage vB_BhaS-171	NC_030904	38975
Bacillus phage phi105	NC_004167	39325
Bacillus phage Pascal	NC_027372	39639
Bacillus phage Pony	NC_022770	39844
Bacillus phage BalMu-1	NC_030945	39873
Bacillus phage Page	NC_022764	39874
Bacillus phage Palmer	NC_028926	40000
Bacillus phage Pavlov	NC_028782	40024
Bacillus phage Pookie	NC_027394	40214
Bacillus virus Wbeta	NC_007734	40867
Bacillus phage BCJA1c	NC_006557	41092
Bacillus phage PBC1	NC_017976	41164
Bacillus phage phi4J1	NC_029008	41486
Bacillus phage BtCS33	NC_018085	41992
Bacillus phage PfEFR-5	NC_031055	43773
Bacillus phage SPP1	NC_004166	44010
Bacillus phage phIS3501	NC_019502	44401
Bacillus phage Waukesha92	NC_025424	45648
Bacillus virus Glittering	NC_022766	49246
Bacillus virus Andromeda	NC_020478	49259
Bacillus virus Curly	NC_020479	49425
Bacillus virus Eoghan	NC_020477	49458
Bacillus virus Riggi	NC_022765	49836
Bacillus virus Finn	NC_020480	50161
Bacillus virus Blastoid	NC_022773	50354
Bacillus phage PM1	NC_020883	50861
Bacillus phage vB_BtS_BMBtp3	NC_028748	51366
Bacillus virus IEBH	NC_011167	53104
Bacillus virus 250	NC_029024	56505
Bacillus phage Stahl	NC_028856	80148
Bacillus phage Slash	NC_022774	80382
Bacillus phage Stills	NC_028777	80798
Bacillus phage Staley	NC_022767	81656

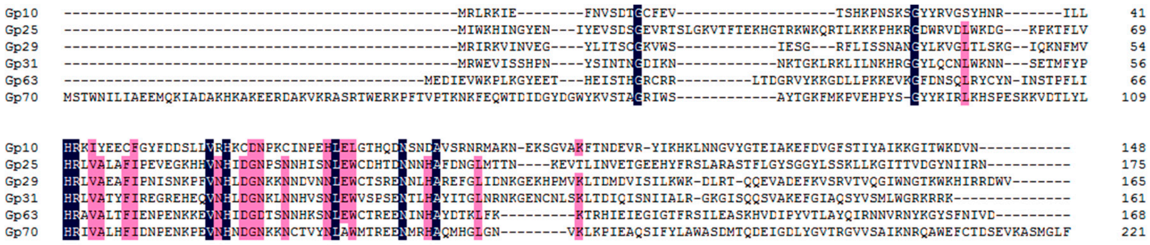

Figure S1 Alignment of the HNH homing endonucleases encoded by phage vB_BthS_BMBphi. The conserve amino acid residues in the six HNH homing endonucleases were shown in color.

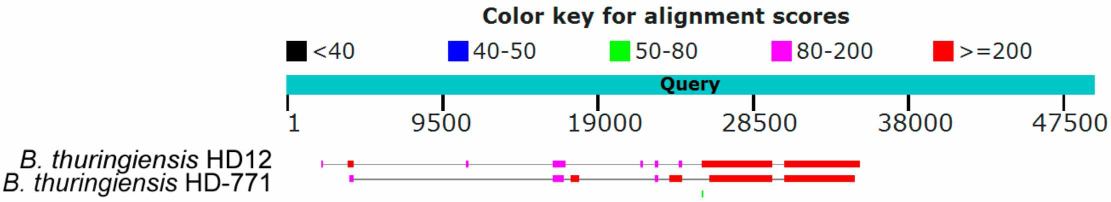

Figure S2 BLASTN analysis of the genome sequence of phage vB_BthS_BMBphi. The genome regions that showed similarity with phage vB_BthS_BMBphi genome were shown and origin organism were indicated.
